# Measuring Population Transmission Risk for HIV: An Alternative Metric of Exposure Risk in Men Who Have Sex with Men (MSM) in the US

**DOI:** 10.1371/journal.pone.0053284

**Published:** 2012-12-28

**Authors:** Colleen F. Kelley, Eli S. Rosenberg, Brandon M. O'Hara, Paula M. Frew, Travis Sanchez, John L. Peterson, Carlos del Rio, Patrick S. Sullivan

**Affiliations:** 1 Department of Medicine, Emory University, Atlanta, Georgia, United States of America; 2 Department of Epidemiology, Rollins School of Public Health, Emory University, Atlanta, Georgia, United States of America; 3 Department of Behavioral Science and Health Education, Rollins School of Public Health, Emory University, Atlanta, Georgia, United States of America; 4 Department of Psychology, Georgia State University, Atlanta, Georgia, United States of America; 5 Hubert Department of Global Health, Rollins School of Public Health, Emory University, Atlanta, Georgia, United States of America; University of Toronto, Canada

## Abstract

**Background:**

Various metrics for HIV burden and treatment success [e.g. HIV prevalence, community viral load (CVL), population viral load (PVL), percent of HIV-positive persons with undetectable viral load] have important public health limitations for understanding disparities.

**Methods and Findings:**

Using data from an ongoing HIV incidence cohort of black and white men who have sex with men (MSM), we propose a new metric to measure the prevalence of those at risk of transmitting HIV and illustrate its value. MSM with plasma VL>400 copies/mL were defined as having ‘transmission risk’. We calculated HIV prevalence, CVL, PVL, percent of HIV-positive with undetectable viral loads, and prevalence of plasma VL>400 copies/ml (%VL400) for black and white MSM. We used Monte Carlo simulation incorporating data on sexual mixing by race to estimate exposure of black and white HIV-negative MSM to a partner with transmission risk via unprotected anal intercourse (UAI). Of 709 MSM recruited, 42% (168/399) black and 14% (44/310) white MSM tested HIV-positive (p<.0001). No significant differences were seen in CVL, PVL, or percent of HIV positive with undetectable viral loads. The %VL400 was 25% (98/393) for black vs. 8% (25/310) for white MSM (p<.0001). Black MSM with 2 UAI partners were estimated to have 40% probability (95% CI: 35%, 45%) of having ≥1 UAI partner with transmission risk vs. 20% for white MSM (CI: 15%, 24%).

**Discussion:**

Despite similarities in other metrics, black MSM in our cohort are three times as likely as white MSM to have HIV transmission risk. With comparable risk behaviors, HIV-negative black MSM have a substantially higher likelihood of encountering a UAI partner at risk of transmitting HIV. Our results support increasing HIV testing, linkage to care, and antiretroviral treatment of HIV-positive MSM to reduce prevalence of those with transmission risk, particularly for black MSM.

## Introduction

Men who have sex with men (MSM) continue to account for the largest risk group in the US for HIV incidence, accounting for 61% of new HIV infections in 2009 [1]. Marked racial disparities seen throughout the HIV epidemic in the US are also present among MSM [2]. Among MSM recruited in venues in 21 US cities, seroprevalence among black respondents was 28% versus 16% among non-Hispanic whites [3]. Examination of differences in individual risk behavior or substance abuse have not explained this disparity; black MSM have lower numbers of casual sex partners, and comparable levels of unprotected anal intercourse (UAI), and drug use [4]. Black MSM do have more sexually transmitted infections, are less likely to be aware of their HIV status, and HIV-positive MSM are less likely to be on anti-retroviral therapy (ART); but differences in incarceration history and circumcision status have not been associated with HIV infection among black and white MSM [4]–[6]. A complete understanding of disparities will allow for the appropriate design and implementation of HIV prevention interventions and is crucial to reduce HIV incidence among MSM.

The National HIV/AIDS Strategy for the United States defines reducing HIV related health disparities as a primary goal and proposes population based metrics, such as HIV incidence and community viral load, as targets for reduction [7]. Population based metrics are essential to understanding the HIV epidemic and to the design, implementation, and evaluation of HIV prevention interventions including behavioral and biomedical prevention interventions. HIV prevalence, the percentage of HIV positive persons divided by the total population, is commonly used to describe the impact of the epidemic on a population and clearly highlights the disparities seen between black and white MSM [2]. The results of the HPTN 052 study that showed a 96% reduction in HIV transmission with the use of ART in serodiscordant, heterosexual couples have energized the public health community around the use of ART as prevention [8], [9] and highlight the need to measure treatment success, represented by low or undetectable viral load, as a benchmark of prevention success.

However, the metrics that are most useful to understand health disparities in HIV transmission are unclear. Community viral load (CVL) [10], the mean [11], [12] or median [13] HIV viral load among those who are aware of their infection and, in some instances, receiving clinical care, and more recently, population viral load (PVL) [10], the mean or median HIV viral load among all HIV positive persons, have been described and proposed as metrics useful to monitor the effect of ART on transmission in a population. In addition, recent data have also been reported on the continuum of HIV care in the United States and the percentage of HIV positive persons who are accessing medical care and who have undetectable viral loads in the US [14], [15].

Although the above metrics provide critical information to understanding the impact of ART as prevention, their utility in understanding disparities in HIV transmission is unclear. Because CVL and PVL concentrate only on those with HIV infection, they may not fully explain disparities in risk of HIV transmission in a given community. For example, it is plausible that two communities could have similar CVL or PVL if similar proportions in each community are on ART but still show disparities in HIV incidence due to underlying differences in HIV prevalence between the two communities. At the same time, if large differences are seen in the continuum of HIV care between two groups and significant disparities exist in the percentage of people with undetectable viral loads, differences in CVL and PVL may be seen but still do not reflect differences in HIV prevalence.

Therefore, in this manuscript, we illustrate the limited utility of these metrics in understanding disparities in HIV transmission in a currently enrolling US cohort of black and white MSM, and propose a new metric to measure the prevalence of those at risk of transmitting HIV. In addition, we demonstrate how this metric can be used to model exposure to HIV in a population by synthesizing information on HIV prevalence, control of viral load, and key behavioral elements. We argue that the prevalence of those at risk of transmitting HIV may be a better prevention intervention target and population metric to reduce disparities in HIV exposure and, ultimately, transmission, particularly among black and white MSM.

## Methods

### Ethics statement

The Institutional Review Board of Emory University approved this study. All participants provided written informed consent prior to enrollment.

### Study Population and Procedures

The *InvolveMENt* study is a currently enrolling, ongoing, cohort study at Emory University designed to examine the individual, dyadic, and community level factors that may contribute to the disparities in HIV and sexually transmitted infection incidence between black and white MSM in Atlanta, Georgia. MSM aged 18–39 years are recruited, regardless of HIV status, from the Atlanta community primarily using time-space venue sampling, with a sampling frame built upon that used in the Atlanta site for the second MSM cycle of the National HIV Behavioral Surveillance System (NHBS) [16]. Facebook was included as a virtual “venue” in the venue sampling frame. Eligible participants are self-identified black and white MSM who report sex with another man in the previous 3 months and who are not in a mutually monogamous relationship, can complete survey instruments in English, live in the Atlanta metropolitan area, are not enrolled in another HIV prevention study, and have no plans to relocate in the subsequent 2 years. Men who self-identified as Hispanic or of other/mixed race were not enrolled. All men, including those who self-report a previous HIV diagnosis, are tested for HIV using a rapid test with confirmatory ELISA and western-blot and complete a detailed computer-assisted self-interview (CASI) questionnaire to evaluate demographic, individual (e.g. number of sexual partners, number of unprotected anal intercourse (UAI) partners, condom use, drug/alcohol use etc.), dyadic (e.g. partner demographics such as age and race, partnership characteristics, etc.), and community level (e.g. poverty, neighborhood violence, etc.) HIV risk. All HIV-positive men, regardless of previous diagnosis, undergo viral load testing (Quest Diagnostics; TaqMan quantitative real-time PCR) and those not already in HIV care are linked to care for further evaluation and treatment as needed. Men who are HIV negative are prospectively followed for up to 24 months and undergo HIV antibody testing at 3–6 month intervals. This report examines baseline visit data for all participants enrolled from July 2010 through June 15, 2012.

### HIV Infection Summary Metrics

HIV prevalence was calculated for all MSM enrolling in the study. Men who were HIV positive at enrollment were considered to be aware of their infection if they reported a previous HIV positive test result on the baseline questionnaire. The proportions of MSM who reported being previously aware of their infection and who reported currently taking ART were calculated for HIV-positive MSM. MSM with HIV plasma viral loads <200 copies/ml were defined as having an undetectable viral load. Men with HIV plasma viral loads >400 copies/ml (%VL400) were defined as having ‘transmission risk’. This viral-load cut-off is a conservative estimate below which HIV transmission is thought to be unlikely based on evidence from discordant heterosexual couples showing limited transmission with plasma viral loads <1500 copies/ml and extremely rare transmission with plasma viral load<400 copies/ml [17], [18] while still allowing for low-level blips in viremia for those on effective ART. Of note, the value of viral load below which HIV transmission is rare is not known for MSM, and it is biologically plausible that this level is lower for MSM than heterosexuals given the differential in transmission probability per exposure event across the rectal mucosa where the majority of transmissions occur among MSM [19], [20]. Therefore, because choosing a viral load cut-off of >400 copies/ml is somewhat arbitrary and given the uncertainty in the appropriate cut-off viral-load, we performed sensitivity analyses defining transmission risk as viral load>50 copies/ml and viral load>1000 copies/ml.

Using this information, empirical distribution functions of viral load and log_10_(viral load), stratified by race, were plotted first among all HIV-positive MSM who were aware of their infection (i.e. the CVL), second among all HIV-positive MSM irrespective of infection awareness (i.e. the PVL), and finally among all MSM participating in the study inclusive of those who were HIV negative (i.e. the transmission risk distribution). These distributions were each compared between black and white MSM using the Kolmogorov-Smirnov Test [21]. For all race-specific distributions, median viral loads were obtained, thus including the traditional CVL and PVL measures, and compared between black and white MSM using the Wilcoxon Rank Sums test. We additionally estimated the proportion of men in each subset who had viral load >400 copies/ml (%VL400), and compared these by race using *X* ^2^ tests.

### HIV Exposure Model

**Figure 1:**
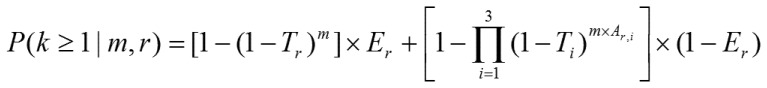
HIV exposure model equation: The estimated the probability of having ≥1 UAI partner with HIV transmission risk (i.e. HIV viral load >400 copies/ml). In this equation, *k* = number of UAI partners with transmission risk (*k*≤*m*); *m* = number of male UAI partners (m = 0 to ∞); *r* = black or white non-Hispanic race/ethnicity (r = 1, 2); *i* = race/ethnicity, same as *r*, and including a third level for ‘other’ non-black or non-white race/ethnicity (*i* = 1,2,3); *T_r_,T_i_* = race-specific %VL400 among male UAI partners; *E_r_* = proportion of participants reporting exclusively same-race UAI partners, among race *r*; *A_r, i_* = the proportion of UAI partners who were race/ethnicity *i*, among participants of race *r* reporting inter-racial UAI partners.

We next sought to understand how the %VL400 in the black and white MSM communities might shape risk of HIV exposure for HIV-negative MSM of each race. This was done with a behavioral model, displayed in Figure 1, which translated this %VL400 estimate from population-level measures of virologic suppression into race-specific probabilities of an HIV-negative individual encountering a UAI partner with transmission risk. This model assumed that the observed race-specific %VL400 distributions of participants also represented the distributions among black and white sex partners. Then, because MSM do not necessarily have racially-concordant partnerships (i.e. assortative racial mixing), we apportioned the race-specific %VL400 to the partners of black and white HIV-negative MSM according to the reported racial composition of their UAI partnerships (E_B_ = 0.71, E_W_ = 0.70, A_B, B_ = 0.34, A_B, W_ = A_B, O_ = 0.33, A_W, W_ = 0.40, A_W, B_ = A_W, O_ = 0.30). The %VL400 for partners of other race/ethnicity (*T_3_*) was calculated as the midpoint between that of black and white non-Hispanics ([*T_1_*+*T_2_*]/2), in accordance with HIV prevalence surveillance estimates [3]. Thus this model used the participants' %VL400 estimates to represent partners, and generated racial-mixing adjusted probabilities of encountering a partner with transmission risk as a function of UAI partner number.

These probabilities were computed and plotted separately for both white and black MSM, for *m* = 0 to 20 UAI partners. We next estimated the variability of these two probabilistic functions by conducting Monte Carlo simulations that allowed input parameters to independently vary according to normal approximations to the binomial distribution. The parameter *T_3_*, described above, was recomputed for each model iteration. Estimates were sorted and used to construct 95% confidence bands around the race-specific probability functions and to conduct hypothesis tests of the racial differences in encountering a partner with HIV transmission risk at fixed partner counts [22]. Specific comparisons were made at the reference points of the 12-month median UAI partner counts for each race among those reporting any UAI, in order to compare the risk among men at greatest behavioral risk for HIV infection. Comparisons were also made at the 12-month median UAI partner counts among the whole sample, for a population-wide perspective of risk. All computations were conducted in SAS 9.3 (Cary, NC).

## Results

Through June 2012, 399 black and 310 white MSM have enrolled into the *InvolveMENt* study. The baseline prevalence of HIV was 42% (95% CI: 37%, 47%) for black MSM and 14% (95% CI: 11%, 19%) for white MSM (p<.0001). Annual HIV incidence among prospectively-followed participants (365 total person-years) was 6.4% for black MSM (10 seroconversions; 95% CI: 3.1%, 11.8%) and 1.0% for white MSM (2 seroconversions; 95% CI: 0.1%, 3.5%). A description of MSM included in this analysis is presented in Table 1. White men in our study were significantly older, had a higher education status, and earned more money than black men. In addition, white men reported significantly more male sexual partners in the previous 12 months and more unprotected anal intercourse than black men.

**Table 1:** Demographic and behavioral characteristics of black and white MSM in the *InvolveMENt* study.

	Black MSM (n = 399)		White MSM (n = 310)		
	*%*	*(total)*	*%*	*(total)*	*p-value* *
**Age** *(years)*		*(n = 399)*		*(n = 310)*	*0.003*
18–19	6.5	(26)	4.5	(14)	
20–24	35.3	(141)	26.1	(81)	
25–29	30.3	(121)	29.0	(90)	
30–39	25.6	(102)	35.2	(109)	
40+	2.3	(9)	5.2	(16)	
**Education**		*(n = 397)*		*(n = 309)*	<.0001
College, post-graduate, or professional school	30.0	(119)	53.4	(165)	
Some college, associate's degree, and/or technical school	44.3	(176)	35.6	(110)	
High school or GED	22.2	(88)	10.4	(32)	
Less than high school	3.5	(14)	0.7	(2)	
**Annual income**		*(n = 369)*		*(n = 304)*	
<$20,000	55.8	(206)	31.9	(97)	<.0001
$20,000–$29,999	15.2	(56)	11.8	(36)	
$30,000–$39,999	14.1	(52)	11.8	(36)	
$40,000–$49,999	6.5	(24)	10.2	(31)	
≥$50,000	8.4	(31)	34.2	(104)	
**Male sex partners, prev. 12 months**		*(n = 397)*		*(n = 308)*	<.0001
1	6.3	(25)	1.6	(5)	
2–5	51.1	(203)	37.0	(114)	
6–10	23.4	(93)	28.6	(88)	
>10	19.1	(76)	32.8	(101)	
**Male unprotected anal intercourse partners, prev. 12 months**		*(n = 394)*		*(n = 306)*	<.0001
0	32.3	(130)	19.3	(59)	
1	24.1	(95)	30.1	(92)	
2–5	33.8	(133)	36.6	(112)	
6–10	4.1	(16)	5.9	(18)	
>10	5.1	(20)	8.2	(25)	

* *The education comparison was made with Fisher's Exact Test. For all other factors, the X^2^ Test was used.*

**Figure 2:**
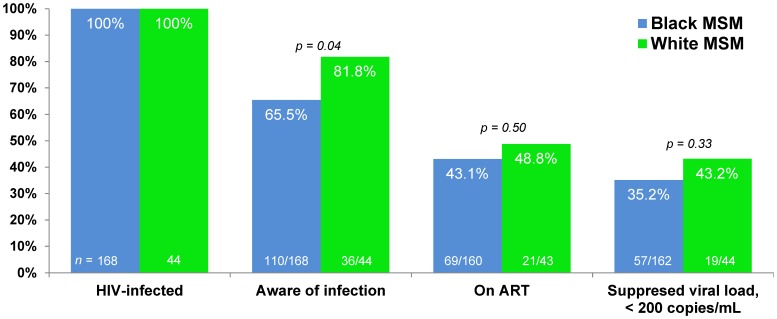
The continuum of HIV care for black and white MSM in the *InvolveMENt* study. Complete data on linkage to and retention in HIV care are not available for this study.

Because differences in access to and engagement in HIV care may differ due to social-demographic differences such as age and income [23], we calculated a continuum of engagement in HIV care similar to that proposed by Gardner et al [14] and the CDC [15] (Figure 2). Complete data on linkage to and retention in care are not available for our cohort. White MSM were more likely than black MSM to report being previously aware of their HIV infection (82% [95% CI: 68%, 91%] vs. 66% [95% CI: 58%, 72%], p = 0.04). No significant differences were seen between black and white MSM in the percent of HIV-positive men who reported currently taking ART or in the percent of men with viral loads <200 copies/ml.

**Figure 3:**
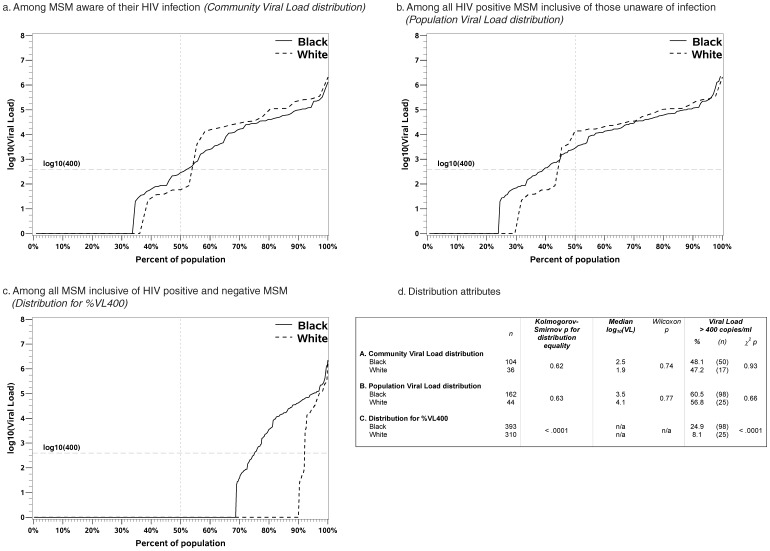
Viral load distribution functions among black and white MSM aware of their HIV infection (Community Viral Load; panel a), among all HIV positive MSM inclusive of those unaware of HIV infection (Population Viral Load; panel B), and among all MSM inclusive of HIV negative and positive (panel c) in the *InvolveMENt* study demonstrating similarities in CVL and PVL due to underlying similarities in the continuum of HIV care. Large differences are evident in the distribution for black and white MSM of MSM at risk of transmitting HIV as this metric accounts for differences in HIV prevalence. Panel D presents the Kolmogorov-Smirnov test for differences in the distributions between black and white MSM for all three distributions, the Wilcoxon Rank Sums test for differences in the median CVL and PVL, and the chi-square test for differences in the prevalence of viral load>400 copies/ml for all three distributions.

Viral load distribution functions for the populations used to calculate the CVL, PVL, and %VL400 are presented in Figure 3. Note that the viral load distribution graphs presented in panel a (CVL) is limited to HIV positive MSM aware of their HIV infection; panel b (PVL) is limited to HIV positive men inclusive of those unaware of their HIV infections; and panel c (%VL400) represents the viral load distribution for all MSM inclusive of HIV negative and positive MSM as defined in the methods. There were no significant differences in the CVL or PVL between black and white MSM. However, there was a large, statistically significant disparity in the distribution and percent of black MSM with HIV transmission risk (%VL400) as compared to white MSM (25% [95% CI: 21%, 29%] vs. 8% [95% CI: 5%, 12%], p<.0001). The percent of all black MSM with HIV viral loads between 2.7–4.0 and 4.0–5.0 log_10_ copies/ml was higher than white MSM (respectively 7% [95% CI: 5%, 10%] vs. 1% [95% CI: 0.02%, 2%], p<.0001; 13% [95% CI: 10%, 17%] vs. 4% [95% CI: 2%, 7%], p<.0001). There was no difference in the proportion of all black MSM with HIV viral loads >5.0 log_10_ copies/ml as compared to white MSM (5% [95% CI: 3%, 7%] vs. 3% [95% CI: 2%, 6%], p = 0.27).

**Figure 4:**
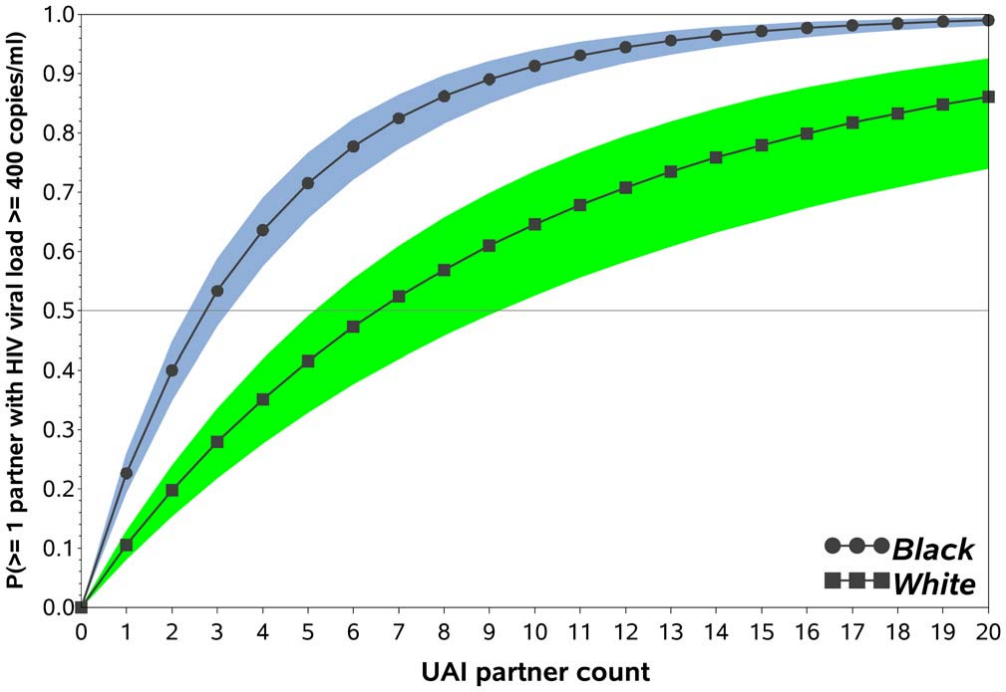
HIV exposure model: The estimated probability of having ≥1 UAI partner with HIV transmission risk (i.e. HIV viral load >400 copies/ml) for black (circles) and white (squares) MSM. Shaded bands represent 95% confidence intervals. This figure demonstrates the differences in HIV exposure based on number of UAI partners between black and white MSM in the InvolveMENt study. This model does is not intended to represent true HIV risk of HIV transmission for a given sexual encounter as necessary covariates such as sexual frequency, sexual practices (i.e. insertive vs. receptive partner), sexually transmitted infections, partner viral load, etc. are not accounted for.

Results of the HIV exposure model are shown in Figure 4, which displays the estimated probability of HIV-negative black and white MSM having at least one sexual partner with HIV transmission risk. This figure is time-scale free and represents the exposure risk accrued across new partnerships: as the number of partners increases, the probability of being exposed to at least one partner with transmission risk increases. For reference, at the median previous 12 month UAI partner count of 1 for black MSM, the estimated probability of having ≥1 UAI partner with transmission risk was 23% (95%: 19%, 26%) and at the median of 2 for white MSM, this was 20% (95% CI: 15%, 24%; p = 0.44). At equal behavioral risk of 2 UAI partners, the median number in the previous 12 months for black and white HIV negative MSM who reported any UAI (p = 0.69), the estimated probabilities were 40% (95% CI 35%, 45%) for black and 20% (95% CI 15%, 24%) for white MSM (p<.0001). In addition, the estimated number of UAI partners to have a 50% chance of having a UAI partner with HIV transmission risk was 3 (95% CI 2, 7) for black and 7 (95% CI 5, 10) for white MSM.

Sensitivity analyses using an alternate transmission risk viral load cut-point of >50 copies/ml yielded prevalence estimates of MSM at risk of transmitting HIV of 30% (95% CI 26%, 35%) for black MSM and 9% (95% CI 6%, 13%) for white MSM (difference p<.0001). At viral load>1000 copies/ml the estimates were 23% (95% CI 19%, 27%) for black MSM and 8% (95% CI 5%, 12%) for white MSM (difference p<.0001). These modest shifts in the percentage of MSM at risk of transmitting HIV relative to those obtained at %VL400 caused no meaningful changes in HIV exposure model results.

## Discussion

Our data show that despite similarities between black and white MSM in CVL, PVL, the percentage of HIV positive men with undetectable viral loads, and significant differences in awareness of HIV infection, black MSM are 3 times as likely as white MSM to have HIV transmission risk. Because of patterns of racial concordance of sexual partnerships, these differences in transmission risk may drive greater risk of HIV exposure for black MSM, despite similar levels of sexual risk behaviors. Although black MSM who have UAI do not have more UAI partners than white MSM, at observed 12-month partner levels, the estimated probability that at least one of those partners will have the potential to transmit HIV for black MSM is over twice that for white MSM. For black MSM, even a relatively low number of UAI partners (e.g. 3) leads to a >50% chance of being exposed to at least 1 partner with the risk of transmitting HIV.

Our results show our new metric provides more insights into disparities in HIV transmission among black and white MSM, compared with CVL, PVL, or the percentage of HIV positive persons who have undetectable viral loads. The %VL400 incorporates both data on HIV prevalence and viral load to better describe HIV exposure, an important driver of HIV transmission, for black and white MSM. Although HIV prevalence estimates are a critical first step in describing disparities, they do not reflect the dynamic nature of transmissibility in a population. At the same time, it must be noted that the large differences in the %VL400 we found are driven primarily by large disparities in HIV prevalence between black and white MSM. However, we feel our measure adds substantially to HIV prevalence measures by reflecting the reduction in HIV transmission seen in those with low or undetectable viral loads due to effective ART [8], [17], [18]. In addition, as increasing numbers of HIV positive persons are on ART, immediate decreases in HIV prevalence will not be seen despite declines in HIV transmission due to reductions in viral load, and additional metrics are necessary to understand ongoing HIV transmission disparities. This may be especially important if factors such as differences in access to care lead to disparities in ART use and viral suppression between populations.

We have shown that CVL and PVL measures are limited in their ability to describe disparities at the population level because they do not also incorporate prevalence data. Therefore, they cannot be used to compare populations with similar engagement in HIV care but different prevalence of HIV infection, such as black and white MSM in our cohort. A strength of our methodology is that it can be applied to other localities or to national data with reliable prevalence estimates to generate population-specific metrics and may be most useful for public health officials in areas of high HIV incidence. For example, if a locale has a reasonable estimate of HIV prevalence and access to viral load data in clinical databases, the prevalence of those at risk of HIV transmission is easily estimated with the assumption that those who are not in clinical care (and thus do not have viral load data available) are at risk of transmitting HIV. In contrast, CVL and PVL require viral load data on all individuals to calculate. In addition, while the CVL has been associated with declines in new HIV diagnoses in areas with high levels of awareness of HIV infection, access to, and engagement in HIV care such as San Francisco and British Colombia [11], [13], this has not been true for areas with lower levels of awareness and engagement in HIV care such as the District of Colombia [12]. Our metric has yet to be associated with HIV incidence, which is a limitation of this analysis and a crucial component of its utility. We do intend future analyses that will include the %VL400 as a predictor of HIV incidence in our ongoing cohort.

The Centers for Disease Control and Prevention has noted that obtaining HIV viral loads on all HIV-positive individuals regardless of their awareness of HIV infection, access to, and engagement in clinical care presents substantial difficulties and is a theoretical measurement at this time [10]. Because our study utilized community-based recruiting and measured HIV viral loads on all MSM, regardless of whether they reported being aware of their HIV infection, we are uniquely able to report population viral load. In addition, our data support the value of including viral load monitoring into HIV surveillance systems that measure HIV prevalence among at-risk groups, such as NHBS, so that estimates of the prevalence of those with HIV transmission risk can be monitored.

In addition to CVL, recent attention has also focused on the percentage of HIV positive individuals with undetectable viral loads and is an important measure used to understand the continuum of HIV care which includes diagnosis of HIV infection, being linked to and retained in HIV care, prescription of ART, and achieving an undetectable viral load [14], [15]. To the extent that black MSM are less likely to be on ART than white MSM, these metrics may be expected to provide useful information in understanding disparities [4], [5]. However, in Atlanta, we show significant differences only in the percent of black MSM who are unaware of their HIV infection as compared to white MSM, and no significant differences in the percent taking ART or with undetectable viral loads. In addition, emerging data from our cohort suggest that self-report of HIV awareness, as was used in this analysis, may not be an accurate measurement of awareness [24] which could explain why we saw differences in HIV awareness but not in the percent with undetectable viral loads. Although our study has limited power to demonstrate differences that may exist in the continuum of HIV care for black and white MSM, these differences are unlikely to fully account for the large disparities in HIV prevalence and incidence. In addition, although focusing on HIV-positive MSM alone may be attractive for HIV prevention interventions [9]; understanding disparities in HIV transmission to HIV-negative MSM using CVL, PVL, and the percent of HIV positive persons with undetectable viral loads will be limited because disparities in HIV prevalence are not incorporated into these metrics.

Behavioral interventions have been shown to reduce occasions of or partners for UAI by 17–27% among MSM [25]. Our model suggests that in order to reduce disparities in HIV exposure between black and white MSM, an extreme reduction in the number of UAI partners for black MSM would be necessary. For example, black MSM with 5 UAI partners have an estimated probability of being exposed to HIV by at least 1 partner of approximately 60%. A highly effective prevention intervention that reduces the number of UAI partners by 1/3 (i.e. to 3.5 UAI partners) will still result in an estimated probability of being exposed to HIV by at least 1 partner of >50%. In contrast, this level of risk is only seen by white MSM with at least seven UAI partners. Because of the minimal impact of even highly effective behavioral interventions on reducing HIV exposure among black MSM, our data support targeting resources to reduce the HIV transmission risk by increasing access, linkage, and retention in HIV care for HIV positive black MSM, supported by behavioral interventions to increase condom use and linkage to and retention in care [26].

There are limitations to these results. First, our sample size is moderate and metrics generated from the *InvolveMENt* cohort study are based on MSM in Atlanta, and may not be generalizable to broader MSM populations. Our sampling methodology, which includes venue-based and internet recruiting, and exclusion of MSM in mutually monogamous relationships limits the generalizability of our results. There is potential for bias in our estimates of CVL, PVL, and %VL400 to the extent that our community-recruited, non-monogamous sample is not adequately representative of all black and white MSM in Atlanta. In addition, we were unable to account for acute HIV infection, which may account for a substantial proportion of HIV incidence in MSM [27], in our estimate of %VL400 as HIV diagnosis was based on antibody testing. However, any bias introduced by this should result in an underestimation of the %VL400 for black MSM as HIV incidence is higher in this group. We recognize that, although %VL400 provides important information beyond that reflected in CVL and PVL, not all health jurisdictions have high-quality prevalence data available to calculate this new metric. In many health jurisdictions where all viral load tests are reportable, calculating CVL using data from surveillance systems may be a more feasible metric, despite its limitations.

We recognize that our model of HIV exposure is demonstrative and does not directly model HIV transmission, for which additional parameters, such as sexual practices (i.e. insertive vs. receptive intercourse, sexual frequency, sexually transmitted infections, partner HIV viral load, etc.) and methods are necessary [26], [28], [29]. It should be noted that the intention is not to model HIV transmission risk but to compare HIV exposure between black and white MSM. Further, this model makes several key assumptions. We do not account for serosorting behavior, which may reduce the likelihood that an HIV-negative man encounters a potentially HIV-transmitting partner, and ultimately the likelihood of becoming infected [30]. Recent reports have found lower levels of pre-sexual discussion of serostatus [31], of serosorting [32], [33], and of the protective value of serosorting [34] among black MSM. To the extent that serosorting may be more common among white MSM, disparities in the probability of encountering a partner at risk of transmitting HIV may be greater than indicated by our model. Although our estimates of racial mixing and partner count are restricted to UAI partners for greatest biological relevance, we have not restricted our %VL400 estimate to men who practice UAI due to sample size limitations.

In conclusion, measuring the prevalence of MSM at risk of transmitting HIV allows incorporation of disparities in HIV prevalence, awareness of infection, and viral load and is a useful tool to monitor HIV exposure, an important driver of HIV transmission disparities. Our example addresses disparities among black and white MSM in Atlanta, but this metric will likely be valuable in understanding disparities between any populations that have differences in HIV prevalence, awareness of infection, and/or engagement in care. CVL and PVL estimates may have limited ability in comparing HIV transmission between two populations. Of particular note, similar reductions in the CVL for black and white MSM will still result in significant disparities in HIV transmission for black MSM. Therefore, resources should be appropriately allocated to dramatically reduce the prevalence of those at risk of transmitting HIV among black MSM by increasing testing, linkage, and retention in HIV care in order to reduce disparities in HIV incidence, supported by coordinated behavioral interventions to increase effectiveness of treatment.
